# The mediating role of achievement motivation in the impact of clinical nurses’ individual-organizational fit on career success

**DOI:** 10.3389/fpsyg.2025.1579410

**Published:** 2025-07-30

**Authors:** Ruishan Liu, Jin Lv, Dehong Meng, Aiqin Hu

**Affiliations:** ^1^Linyi Maternal and Child Health Hospital, Linyi, China; ^2^Department of Nursing, The First Affiliated Hospital of Shandong First Medical University (Qianfoshan Hospital of Shandong Province), Jinan, China; ^3^Linyi Hospital of Traditional Chinese Medicine, Linyi, China

**Keywords:** clinical nurses, personal-organizational fit, achievement motivation, career success, mediating effect

## Abstract

**Introduction:**

This study investigates the mediating role of achievement motivation in the relationship between nurses’ personal-organizational fit and their perception of career success. Additionally, it examines how the dimensions of pursuing success and avoiding failure within achievement motivation influence nurses’ career success perception.

**Method:**

A cross-sectional online survey was conducted, with 366 nurses from Linyi City serving as research participants between August and November 2024. Data were collected via a structured questionnaire and analyzed using Amos and SPSS statistical software.

**Results:**

(1) Personal-organizational fit exhibited a significant positive correlation with nurses’ career success perception. (2) The motive of avoiding failure negatively influenced career success perception, whereas the motive of pursuing success positively impacted it. (3) After controlling for demographic variables such as gender, age, education level, and professional title, both pursuing success and avoiding failure motives significantly mediated the relationship between personal-organizational fit and career success perception.

**Discussion:**

Clinical nurses demonstrate a moderate level of career success perception. Nursing managers can foster a harmonious work environment to enhance clinical nurses’ achievement motivation, thereby improving their career success perception. Practical strategies include enhancing personal-organizational fit, strengthening the motive of pursuing success, and mitigating the motive of avoiding failure, which can directly or indirectly elevate nurses’ career success perception.

## 1 Introduction

The global shortage of nurses underscores the critical importance of addressing their career development needs and providing more opportunities for advancement in their professional roles (Afsar and Masood, 2017). The annual turnover rate of nurses in China exceeds 2.15% (Liu et al., 2024). The professional challenges faced by Chinese nurses exhibit cultural specificity, including: (1) the hierarchical structure within hospitals limits professional autonomy, and (2) the tension between family and career is more pronounced in a collectivist cultural context. These factors underscore the unique significance of researching the professional success mechanisms of clinical nurses in China. Haibo et al. (2018) reported that employees with a strong sense of career success exhibit greater happiness and job satisfaction, along with a reduced intention to quit. The concept of career success refers to the positive psychological experiences and feelings of achievement that individuals derive from their work environments (Chen, 2019). For nurses, the sense of professional success arises from the continuous accumulation of nursing experience and the subjective perception of accomplishment associated with engaging in professional nursing practice (Dan et al., 2018). Evaluation criteria for career success primarily encompass both subjective and objective dimensions: traditional indicators of objective career success include salary levels, job rank, and the number of promotions, among others. Subjective career success mainly involves job satisfaction, work fulfillment, and self-assessment of job performance (Asghari et al., 2021). As a key goal of career development, the sense of career success plays a pivotal role in reducing nurse burnout, enhancing retention intentions, improving patient care quality, stimulating innovative behaviors among nurses, and advancing the nursing profession as a whole (Liu and Liu, 2016). Consequently, it is imperative to investigate the predictive factors and mechanisms underlying the sense of career success among nurses to provide a reference framework for strategies aimed at fostering their career development.

### 1.1 The relationship between person-organization fit and nurses’ sense of career success

In recent years, among the myriad factors influencing career success, work environment factors have garnered increasing attention from researchers (Wang et al., 2019). Person-organization fit, also referred to as person-organization alignment, primarily describes the degree of congruence between an individual and their organization in terms of culture, values, and other attributes. It highlights the influence of the work atmosphere generated through the interaction between the individual and the organization on the individual’s experiences and outcomes. Cheng and Wang (2017) posits that the work environment significantly impacts nurses’ sense of career success and suggests that managers can enhance this sense by improving the work atmosphere. Foreign scholars such as Bailout (2007) argue that the primary determinants of career success are the alignment between personal characteristics and the work environment. Furthermore, the person-environment fit theory emphasizes that the interaction between an individual and their environmental context influences their attitudes and behaviors (Luo and Chen, 2021). Based on the aforementioned literature and theoretical frameworks, it can be inferred that: person-organization fit → career success.

### 1.2 The mediating role of achievement motivation

Career success can be influenced by external work environment factors and one’s own personal factors. Therefore, merely exploring the direct relationship between work environment factors, such as individual-organizational fit, and career success reveals little about the significance of intervention practices. Efficient intervention programs must reveal how the relationship between individuals and organizations affects nurses’ career success through empirical research. Social cognitive theory suggests that the external environment impacts an individual’s psychological environment, ultimately influencing his or her behavior and outcome (Bandura, 2001). Achievement motivation is the intrinsic drive for self-improvement and the pursuit of success. It emphasizes the individual’s control of the environment and realization of their potential (Waluyohadi, 2019). Jong and Goede (2015) suggested that the degree of fit between an individual and an organization impacts an individual’s achievement motivation. Meanwhile, Miao et al. (2020) showed that achievement motivation plays a key role in professional development in high-stress clinical environments. Liu et al. (2013) showed that the level of achievement motivation affects an individual’s sense of career success. These findings suggest that motivation may be an important mediator of organizational matching in influencing career outcomes. Based on these theories and literature, this study posits that achievement motivation is a significant mediator between individual-organizational fit and career success. In other words, individual-organizational fit leads to achievement motivation, which leads to nurses’ career success. It can be inferred that: person-organization fit → achievement motivation → career success.

### 1.3 Research hypothesis model

In summary, this study intends to examine the mediating role of achievement motivation in the relationship between personal-organizational fit and nurses’ perceived career success, and to construct a theoretical model that integrates social cognitive theory and the above research hypotheses. We propose that personal-organizational fit (environmental factors) influences perceived career success (behavioral outcomes) by shaping nurses’ achievement motivation (personal agency). Specifically, when nurses perceive that their values are aligned with the organization (environment), it enhances their self-efficacy to pursue career goals (agency), which in turn enhances career satisfaction (outcome). This framework supports our hypotheses that (1) individual-organizational fit positively predicts career success, and (2) achievement motivation mediates this relationship through the dual paths of ‘pursuit of success’ and ‘avoidance of failure’.

**Figure 1:**
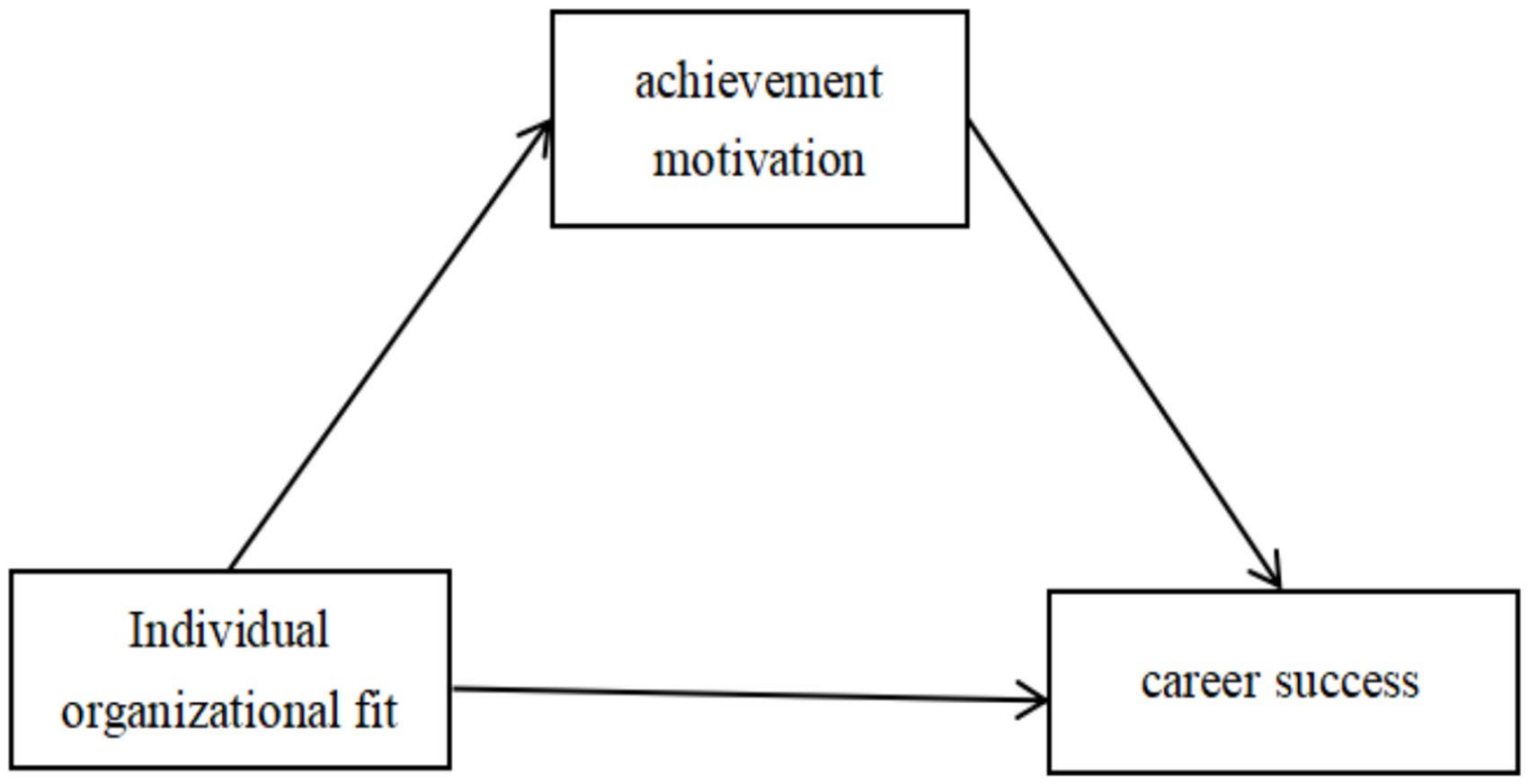
Studies the hypothetical model.

Figure 1 visualizes the hypothesized model for this study.

## 2 Materials and methods

### 2.1 Participants

From August to November 2024, nurses from three tertiary general hospitals in Linyi City were chosen as study participants using the convenience sampling method. Exclusion criteria include: ① nurses on maternity and sick leave during the survey period; ② registered nurses practicing clinical nursing; ③ working experience ≥1 year; ④ all nurses provided informed consent and willingly participated in this study; ⑤ nurses engaged in rotational assignments and advanced academic pursuits. The sample size was estimated using G*Power 3.1 software with *α* = 0.05 and a medium effect size *d* = 0.5. The results indicated that the minimum sample size was 276. Considering a 20% dropout rate, the final sample size included was 366. The hospital ethics committee reviewed this study (QTL-YXLL-2024097).

### 2.2 Instrument

#### 2.2.1 General information questionnaire

It had thirteen entries and was created by the researcher after she reviewed the literature. The following were listed: department, years of service, title, position, monthly income, number of night shifts, gender, age, marital status, number of children, highest degree of education, and reasons for selecting nursing, motional exhaustion, work environment, job role complexity, or leadership style.

#### 2.2.2 Career Success Scale (CSS)

The Career Success Scale of (Bandura, 2001) as amended by Li (2020), was used in this study. The scale was compiled concerning the career satisfaction measurement scale of Greenhaus, a foreign scholar, and combined with China’s national conditions. In four dimensions—career advancement, independence and happiness, recognition, and relationship network—it has twenty-one entries. The score spans from 21 to 105 points on Likert’s 5-point scale (1 being special non-conformity and 5 being particular conformance); the higher the score, the more successful the nurses feel in their careers. The scale’s Cronbach’s alpha coefficient in this study is 0.978.

#### 2.2.3 Personal-Organizational Fit Scale (POFS)

The Individual-Organizational Fit Scale, created by Xiang (2019) specifically for nurses, was utilized in this study. It has 40 items in three dimensions: values fit, individual needs-organizational supply, and individual competence-organizational needs. Higher ratings indicate a stronger personal-organizational fit for nurses. The Likert scale has a score range of 40–200, with 5 representing “very highly valued” and 1 representing “not valued.” In this investigation, the scale’s Cronbach’s alpha coefficient was 0.981.

#### 2.2.4 Achievement Motivation Scale (AMS)

The Achievement Motivation Scale used in this study was revised by our scholars Renmin and Kunta (1992) based on our national conditions. It is divided into two dimensions, the motivation to pursue success and the motivation to avoid failure, with 30 questions, and adopts the Likert 5-point scale (1 = completely non-compliant-5 = completely compliant). The scale is as follows: total motivation to pursue success and motivation to avoid failure, with a total score range of −45 to 45 points, with higher scores representing higher levels of achievement motivation. The Cronbach’s alpha coefficient for this scale in this study was 0.793.

### 2.3 Data collection methods

This study collected samples through the WeChat network channel using the Wenjuanxing platform. The first page of the questionnaire clearly stated the purpose, significance of the survey and the precautions for filling it out. After obtaining informed consent, nurses voluntarily scanned the QR code or clicked the link to complete the filling. All items in the questionnaire were set as required questions, and each IP address was only allowed to fill it out once to ensure the uniqueness and reliability of the data. Only after completing all the questions could the questionnaire be submitted anonymously. A total of 398 questionnaires were distributed in this study. After excluding 32 invalid responses—defined as those with patterned answering or a completion time of less than 2 min—366 valid samples were retained, yielding a response rate of 92%. To further justify the sample adequacy, a statistical power analysis was conducted using G*Power software. The results indicated that with a medium effect size (effect size = 0.30), a significance level of *α* = 0.05, and a desired statistical power greater than 0.80, the sample size of 366 was sufficient to meet the requirements for structural equation modeling, demonstrating robust statistical power.

## 3 Statistical processing

SPSS 23.0 was used for statistical analysis. A *t*-test or F-test was used to perform the measurement statistics, which were then reported as $\bar{x}$ ± *s*. The *X*^2^ test was used to express count data as rate/percentage. With nurses’ career success score as the dependent variable, Pearson correlation analysis was used to examine the relationship between their personal-organizational fit, achievement motivation, and career success. Multivariate linear stepwise regression analysis was used to examine the statistically significant factors in the univariate analysis, while the Bootstrap method was employed to verify the three relationships using the SEM structural equations created by Amos 21.0. Psychological consistency’s mediating role was examined using the relationship and bootstrap approaches.

## 4 Results

### 4.1 Results on career success, achievement motivation, and individual-organizational fit dimensions

According to the findings, nurses’ overall mean score for personal-organizational fit was 106.08 ± 36.87; their overall mean score for accomplishment motivation was 1.79 ± 1.59. More people were motivated to succeed (59.57 ± 12.93) than to fail (32.66 ± 14.38). The mean score for nurses’ overall perception of accomplishment in their careers was 17.90 ± 4.96. Details are in Table 1.

**Table 1:** Scores on the dimensions of individual-organizational fit, achievement motivation, and career success.

Variant	Entry	Actual score	Entry parity (accountancy)	Upper quartile
Individual-organizational fit	40	106.08 ± 36.87	2.62 ± 0.92	2.68
Values fit	22	51.74 ± 21.01	2.35 ± 0.96	2.14
Individual-demand organization supply	10	26.35 ± 12.21	2.64 ± 1.22	2.40
Individual-competence organizational needs	8	15.84 ± 7.37	1.97 ± 0.927	1.86
Achievement motivation	30	1.79 ± 1.59	0.12 ± 0.11	0.12
Motivation for success	15	59.57 ± 12.93	3.97 ± 0.86	4.10
Motivation to avoid failure	15	32.66 ± 14.38	2.18 ± 0.96	1.97
Sense of professional success	21	68.31 ± 12.94	3.25 ± 0.62	3.20
Career progression	5	17.90 ± 4.96	3.58 ± 0.99	3.40
Free and happy	5	17.28 ± 5.91	3.46 ± 1.18	3.50
Accreditation	5	18.55 ± 4.81	3.70 ± 0.97	3.67
Network	6	21.18 ± 5.91	3.50 ± 1.03	3.40

### 4.2 Evaluation of the findings from the one-way comparison of the general data of various research participants

A one-way analysis of variance was employed to compare the career success scores of nurses based on varying general information. The findings indicated that variations in age, marital status, service modality, years of experience, title, administrative role, motivation for selecting nursing, and monthly night shifts were statistically significant when comparing nurses’ scores related to career success (all *p* < 0.05). Refer to Table 2 for specifics.

**Table 2:** Comparison of different nurses’ career success scores for general information (*N =* 366).

Norm	Number of examples	Career success score	*t/F*	*P*
Distinguishing between the sexes			1.902^a^	0.058
Male	37	72.14 ± 11.42		
Daughter	329	67.88 ± 13.04		
(A person’s) age			24.12^b^	0.017*
20–30 years old	269	67.80 ± 12.61		
31–40 years	74	67.89 ± 13.06		
>40 years old	27	75.74 ± 14.63		
Marital status			6.31^b^	<0.001**
Unmarried	185	67.71 ± 12.64		
Married	177	68.66 ± 12.73		
Divorced/widowed	4	57.00 ± 12.73		
Number of children			1.042^b^	0.347
0	210	68.31 ± 12.89		
1	99	67.04 ± 13.73		
2	54	70.24 ± 11.60		
≥3	3	75.67 ± 10.07		
Highest level of education			0.892^b^	0.411
Specialized training school	72	68.39 ± 12.63		
Undergraduate (adjective)	236	67.80 ± 13.37		
Graduate students and above	8	70.33 ± 11.44		
Mode of appointment			4.14^b^	0.003*
Contract system	196	67.68 ± 12.40		
Authorized strength	102	70.56 ± 13.26		
Labor dispatch	10	59.90 ± 11.50		
Personnel agent	18	60.72 ± 11.10		
(sth. or sb) else	40	71.23 ± 13.83		
Section			1.306^b^	0.410
General medicine	99	69.48 ± 12.83		
Neurosurgery	63	70.46 ± 12.15		
Department of gynecology and obstetrics	13	66.69 ± 9.18		
Gynecology	17	62.35 ± 10.76		
Emergency call	48	31.92 ± 6.29		
ICU	9	70.78 ± 19.24		
Operating rooms	16	66.38 ± 9.14		
Outpatient service	26	67.08 ± 10.40		
(sth. or sb) else	76	68.15 ± 15.30		
Years of experience			6.002^b^	0.001*
1–5 years	237	67.65 ± 12.67		
6–10 years	73	68.71 ± 12.76		
11–15 years	24	69.33 ± 11.57		
>15 years	30	76.87 ± 13.68		
Title			3.587^b^	0.014
Physiotherapists	186	66.21 ± 12.78		
Physiotherapists	96	66.84 ± 12.02		
Nurse practitioner-in-charge	71	68.58 ± 14.13		
Associate Nurse Practitioner and above	13	79.23 ± 10.76		
Administrative position			−3.908^a^	<0.001**
Clinical nurse	344	67.66 ± 12.67		
Nursing managers	22	78.55 ± 13.10		
Monthly income ($)			0.309^b^	0.819
<7,000	235	68.37 ± 12.89		
7,000–10,000	84	68.74 ± 14.22		
10,001–15,000	38	66.61 ± 11.27		
>15,000	9	70.11 ± 8.25		
Reasons for choosing care			8.350^b^	<0.001**
Personal preference	109	74.67 ± 12.58		
Parental views	88	65.28 ± 13.13		
Referrals	14	65.79 ± 13.86		
Good luck finding a job	58	64.64 ± 9.26		
School transfer	89	66.29 ± 11.99		
(sth. or sb) else	66	65.47 ± 11.82		
Number of night shifts per month			4.36^b^	0.003*
Not have	62	71.15 ± 13.93		
≤4	41	70.20 ± 13.82		
≤8	174	69.20 ± 12.51		
>8	88	64.14 ± 11.85		

a Indicates *t*-value.

b Indicates *F*-value.

*Stands for *p* < 0.05; **Stands for *p* < 0.001.

### 4.3 Correlation analysis of individual-organization fit, achievement motivation, and career success

The study’s findings indicated a substantial positive link between nurses’ personal-organizational fit and their motivation for achievement and professional success (*r* = 0.613, *r* = 0.548, *r* = 0.673, all *p* < 0.001). A substantial negative association existed between nurses’ personal-organizational fit and their drive to avoid failure and achieve career success (*r* = −0.508, −0.521, −0.527, all *p* < 0.001). 0.521, −0.527, all *p* < 0.001. Refer to Table 3 for specifics.

**Table 3:** Correlation matrix of individual organizational fit, career success and achievement motivation (*N* = 366, *r*).

Meters	Individual-organizational fit	Sense of professional success	Motivation for success	Motivation to avoid failure
Individual-organizational fit	1			
Sense of professional success	0.613**	1		
Motivation for success	0.548**	0.673**	1	
Motivation to avoid failure	−0.508**	−0.521**	−0.527**	1

**Represents *p* < 0.001.

### 4.4 Multifactorial regression analysis of nurses’ career success

The sense of career success was designated as the dependent variable, while all statistically significant general information from the univariate analysis was classified as the independent variable. Additionally, personal-organizational fit and accomplishment motivation were incorporated for multiple linear stepwise regression analyses. The findings indicated that personal-organizational fit, job position, motivation for achievement, reasons for selecting nursing, and the frequency of night shifts per month significantly impacted clinical nurses’ perception of career success in tertiary general hospitals (*p* < 0.05). These factors accounted for 34.4% of the total variance, with personal-organizational fit exhibiting the highest standard regression coefficient, establishing it as the primary predictor of nurses’ career success perception. Refer to Table 4 for specifics.

**Table 4:** Multiple linear stepwise regression analysis of nurses’ career success.

Implicit variable	Independent variable	Non-standardized coefficient		Standardized coefficient	*t*	*p*	95% *BootCI*
		*B*	Standard error	*β*			
Sense of professional success	(Constant)	79.719	3.407	–	23.402	<0.001	
	Individual-organizational fit	−0.179	0.016	−0.509	−11.305	<0.001	−0.188 ~ −0.130
	Duties	9.651	2.397	0.178	4.026	<0.001	0.223 ~ 0.416
	Achievement Motivation	1.226	0.364	0.151	3.370	0.001	3.883 ~ 12.832
	Reasons for choosing care	−0.855	0.319	−0.122	−2.684	0.008	−0.676 ~ −0.048
	Number of night shifts per month	−1.282	0.576	−0.098	−2.226	0.027	−0.191 ~ −0.131

*R* = 0.587, *R^2^* = 0.344, Adjusted *R^2^* = 0.335, *F* = 37.453, *p* < 0.05. Predictor variables: individual-organizational fit, job title, motivation to succeed, reasons for choosing nursing, number of night shifts per month.

### 4.5 Common method bias (CMB)

The common method bias test was conducted using the Harman single-factor test method. The results showed that there were 18 factor characteristic roots greater than 1. Among them, the variance explanation rate of the first factor was 33.25%, which was less than the critical value of 40%. Therefore, there was no common method bias problem in this study.

## 5 Structural equation modeling examination of the mediating effect of achievement motivation on the relationship between nurses’ personal-organizational fit and career success

**Figure 2:**
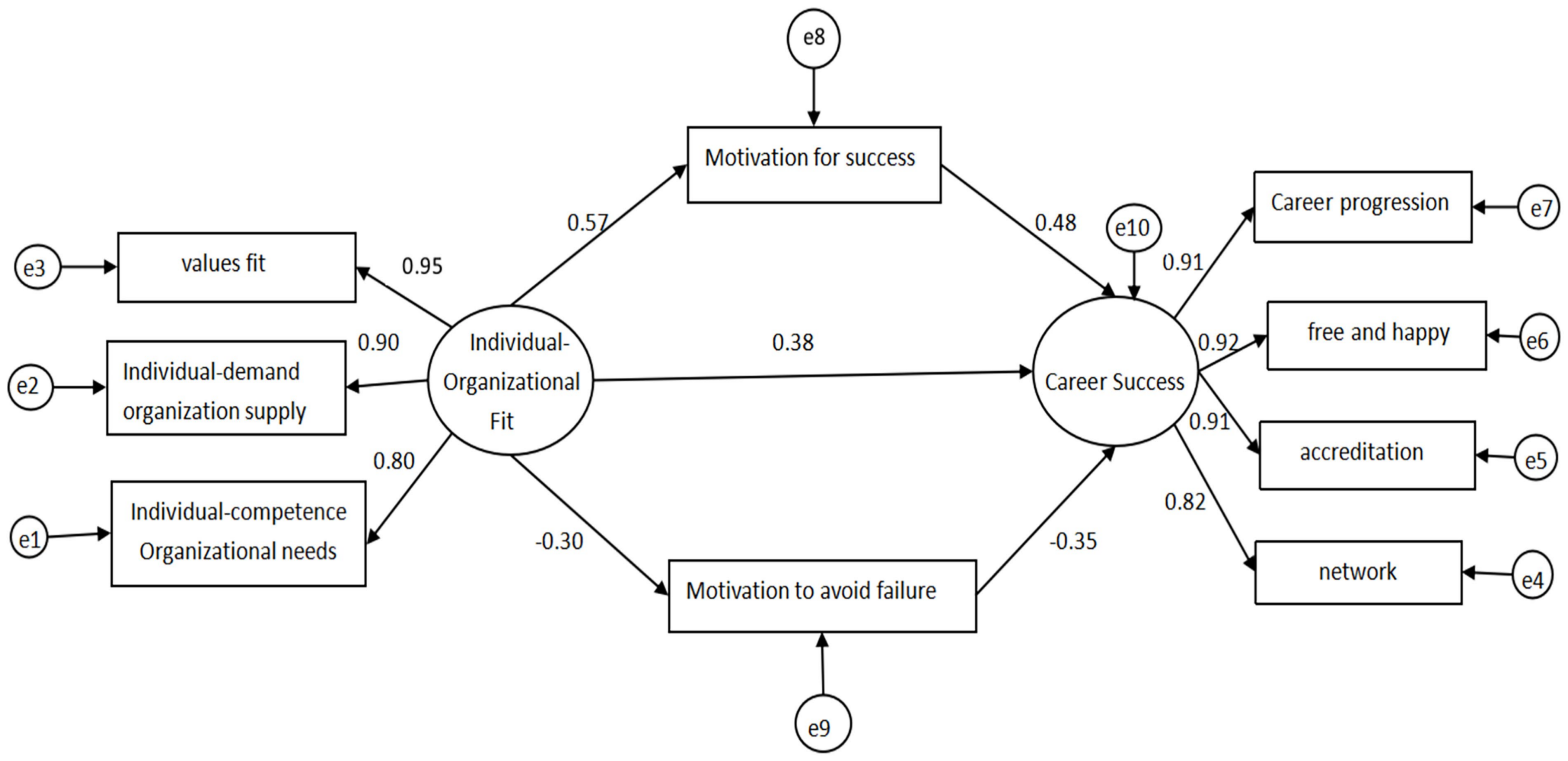
The mediating role of achievement motivation in the impact of clinical nurses’ individual-organizational fit on career success.

Utilizing the correlation and regression analyses among various variables, a hypothetical model was formulated: the nurses’ personal-organizational fit serves as the independent variable, the sense of professional success functions as the dependent variable, with the motivation to pursue success acting as the mediator variable for Path 1, and the motivation to avoid failure serving as the mediator variable for Path 2. The findings of the investigation are presented in Figure 2: all primary fitting indices of the model fall within the allowed range, indicating a well-fitted model (Table 5). The coefficients for each path are specified in Table 6.

**Table 5:** Structural equation model fitting metrics.

Sports event	*x*^2^/*df*	GFI	AGFI	IFI	CFI	TLI	RMSEA
Standard of judgment	*<5*	*>0.90*	*>0.90*	*>0.90*	*>0.90*	*>0.90*	*<0.08*
Verification results	*2.350*	*0.965*	*0.934*	*0.988*	*0.988*	*0.982*	*0.061*

*x*^2^/*df*, Chi-square to degrees of freedom ratio; GFI, Goodness of fit index; AGFI, Adjusted goodness of fit index; IFI, Incremental fit index; CFI, Comparative fit index; TLI, non-normed fit index; RMSEA, root mean square error of approximation.

**Table 6:** Number of paths for nurses’ personal organizational fit-achievement motivation-career success.

Trails	Regression coefficient	Standardized regression coefficient	*SE*	*CR* value	*p*
Path 1					
Individual organizational fit → motivation for success	0.663	0.502	0.058	11.334	<0.001
Motivation for success → professional success	0.498	0.476	0.048	10.294	<0.001
Individual organizational fit → career success	0.468	0.384	0.60	7.771	<0.001
Path 2					
Individual organizational fit → motivation to avoid failure	−0.388	−0.300	0.081	−4.93	<0.001
Motivation to avoid failure → sense of professional success	−0.369	−0.347	0.065	−5.636	<0.001
Individual organizational fit → career success	0.468	0.384	0.60	7.771	<0.001

The bootstrap method, involving 5,000 repetitions, was utilized to assess the mediating effect. The findings indicated that the 95% confidence intervals for the total, direct, and indirect effects of Model 1 and Model 2 did not encompass 0, suggesting that achievement motivation partially mediates the relationship between nurses’ personal-organizational fit and career success. Refer to Table 7 for specifics.

**Table 7:** Bootstrap test of individual-organization fit on career success.

Effect (scientific phenomenon)	Efficiency value	*SE*	95% *BootCI*	*p*	Effect proportion
Path 1					
Total effect	0.65	0.553	0.710 ~ 0.928	<0.001	
Direct effect	0.38	0.057	0.355 ~ 0.579	<0.001	58.46%
Indirect effect	0.27	0.042	0.270 ~ 0.495	<0.001	41.54%
Path 2					
Total effect	0.49	0.553	0.710 ~ 0.928	<0.001	
Direct effect	0.38	0.061	0.508 ~ 0.749	<0.001	77.55%
Indirect effect	0.11	0.046	0.105 ~ 0.284	<0.001	22.44%

## 6 Discussions

### 6.1 Present condition of individual nurses’ organizational alignment, accomplishment motivation, and professional success

The results of this survey show that the personal-organizational fit score of nurses in tertiary hospitals in Linyi City is at a medium level, and it is higher than that of Gao and Zheng (2022) in their latest survey of nurses in various hospitals in Beijing. One possible reason for this is regional management differences. As a pilot of Shandong Province’s healthcare reform, Linyi’s tertiary hospitals likely offer clearer career paths and performance incentives than overloaded hospitals in the Beijing metropolitan area. Among them, the score of the personal-demand organizational-supply dimension is the highest. This might be because the nurses in this study all come from tertiary general hospitals in Linyi City, and the hospitals have strong comprehensive strength, which can provide good salary benefits, as well as opportunities for further training and continuing education for the nurses, facilitating their acquisition of professional satisfaction and self-growth from the organization. The score of the personal-capability organizational-demand dimension is the lowest, which might be because the majority of the nurses in this study have less than 5 years of work experience. Their comprehensive assessment and comprehensive analysis abilities for patients, professional theoretical knowledge, practical abilities, stress handling abilities, and nurse–patient communication abilities need to be improved. It is suggested that nursing managers should regularly provide reinforcement training for junior nurses in terms of theoretical knowledge and operational skills, cultivate their ability to deal with difficulties, and build their confidence in solving problems (Yang et al., 2021). The score of the achievement motivation is at a medium level. The score of the pursuit of success motivation is higher than that of avoiding failure, which is different from the research results of Zhu and Kuang (2022) on newly recruited nurses. Thus, it can be seen that with the accumulation of work experience, nurses have a more comprehensive understanding of their own abilities. After successfully handling various nursing problems many times, their self-efficacy will be enhanced. This self-efficacy will make them believe that they have the ability to achieve more success and have less concern and worry about failure, and thus are more inclined to pursue success. The score of career success is at a medium level. Although it is higher than those of Liu et al. (2019), Kong et al. (2020), Wu et al. (2021), and Cheng and Wang (2017) in previous studies, there is still some room for improvement. The score of the recognition dimension is higher than other dimensions. The reason for this might be that since the normalization of the epidemic, nurses have taken on the main task of citizen nucleic acid testing, and have received respect and recognition from the public, communities, and health departments, which has greatly improved the social status of nurses.

### 6.2 Examination of elements affecting nurses’ perception of professional achievement

Multiple linear regression study indicated that position, motivation for selecting nursing, and frequency of night shifts per month were the primary determinants of nurses’ career success based on demographic information. This study demonstrated that nursing managers exhibited superior career success scores compared to general nurses, attributable to several factors: nursing managers possess advanced theoretical knowledge, scientific research capabilities, professionalism, and operational effectiveness; their career value is acknowledged by patients and other stakeholders; they experience greater satisfaction with their social status and remuneration; and they derive fulfillment from their work. Their scores for career success are likewise elevated. Consequently, it is advisable for nursing managers to enhance the training of clinical nurses in scientific research and innovation capabilities, while developing personalized and targeted training programs based on the varying competencies of nurses and their respective career stages. Additionally, hospital administration should refine the career advancement system and implement favorable policies regarding administrative and title promotions to bolster nurses’ competitiveness and sense of professional achievement.

A negative link exists between the frequency of night shifts each month and career success; specifically, an increase in night shifts correlates with a decrease in the career success of nurses. This may result from the limited number of night shift nurses relative to their day shift counterparts; the task is substantial, the body possesses greater physical strength and stamina, and it endures prolonged states of overload. Simultaneously, repeated night shifts disrupt the body’s circadian rhythm, resulting in diminished sleep quality and immunity among nurses, which significantly impacts their physical and emotional well-being. The frequency of night shifts for nurses in this survey predominantly ranges from 5 to 8 days. Consequently, it is advisable for nursing managers to judiciously schedule night shift frequency, refine the shift system, and augment the off-duty time and performance of nurses frequently assigned to night shifts, in order to mitigate occupational stress and fatigue, while also enhancing their work motivation and sense of achievement.

Individuals who deliberately select the nursing profession are more inclined to experience a sense of job accomplishment than those who passively enter the field for alternative reasons. Nurses who deliberately select the nursing profession are likely more inclined to invest time and effort in refining their professional skills and knowledge to augment their competitive edge. Many nurses who choose the profession for extrinsic reasons lack a comprehensive comprehension of it. Upon entering this field, the disparity between ideal and reality is substantial, rapidly engendering a detrimental inferiority complex or even resistance, so impacting one’s perception of professional accomplishment. Consequently, it is recommended that nursing managers proactively implement nursing-related professional education initiatives to cultivate positive professional attitudes and values among nurses; concurrently, they should promote the professional image of nurses to the public, elevate the social status of nurses, and enhance their sense of identity and job satisfaction.

### 6.3 The correlation among individual-organizational fit, achievement motivation, and career success is significant

This study identified a significant association between individual-organizational fit and career success; as individual-organizational fit increases, so does career success. This may occur when nurses establish a sense of shared purpose with their organizations and attain a significant alignment, motivating them to confront future challenges to achieve the mutually beneficial objective of career advancement and organizational performance enhancement. The person-environment fit theory posits that a strong alignment between individual values, aspirations, or ambitions and their environment can enhance mental health outcomes (Zhu and Kuang, 2022). A positive mindset fosters nurses’ professional success. Secondly, individual-organizational fit exhibited a positive correlation with the incentive to achieve success and a negative correlation with the urge to evade failure. This suggests that when nurses align closely with their business, they perceive challenges as motivation and are inclined to proactively address obstacles and strive for success. Conversely, when nurses exhibit a low alignment with the organization, they experience negative emotions characterized by avoidance and withdrawal in response to failure, resulting in diminished accomplishment motivation. The aforementioned findings should capture the interest of hospital administrators, who ought to facilitate an optimal alignment between nurses and organizations as a foundational step, cultivate a favorable macro and micro environment, enhance the hospital welfare and human care systems, promote harmonious relationships among colleagues and between doctors and patients, and implement a multifaceted strategy to effectively elevate the achievement motivation and sense of professional success among nursing personnel (Luo and Chen, 2021).

### 6.4 The mediating role of achievement motivation in the individual-organizational fit of nurses and career success

The social cognition theory posits that the three factors—the external environment, personal emotions, and individual behavioral intentions—interact with and influence each other (Zhang and Li, 2024). Based on this theory, it can be inferred that career success perception is not only influenced by external work environment factors, but also by internal factors. The results of the structural equation model in this study also confirm that achievement motivation plays a partial mediating role between the nurse’s personal-organizational fit and career success perception. This indicates that the degree of match between nurses and the organization may directly affect their career success perception, or it may indirectly affect career success perception through achievement motivation. Conversely, when nurses experience frustrating integration with the organization, they feel anxious and apprehensive, leading them to purposefully evade challenges to prevent failure. They diminish their enthusiasm for their profession, hence reducing their perception of career success. Nursing managers are advised to acknowledge the mediating function of achievement motivation in enhancing nurses’ career success. The stronger mediating effect of success-seeking motivation aligns with the achievement-oriented nature of Chinese nursing culture. The Confucian value of self-improvement makes pursuing positive goals a better predictor of career outcomes than avoiding risk. This contrasts with research on corporate employees (Luo and Chen, 2021). Hospital administrators are advised to enhance nurses’ personal-organizational alignment by bolstering the cultural attractiveness of nurse leaders, systematically scheduling departmental gatherings, implementing pre-service training for new nurses, and executing hierarchical management frameworks (Zhao et al., 2025). By cultivating a transformational leadership style, fostering a harmonious organizational climate, establishing a development platform, and enhancing nurses’ core competencies to elevate their motivation for success, nursing personnel can thrive in a positive and healthy work environment, thereby attaining professional success (Ding et al., 2022).

## 7 Conclusion

This study is the first to adopt a Chinese personal-organization fit measurement specifically designed for nursing staff, and it explores the relationship between this fit and career success, as well as the mechanism pathways. It also expands the research scope of the person-environment matching theory and social cognitive theory, emphasizing the influence of individual psychological factors on career success, providing reference and development directions for nursing managers to enhance nurses’ career success.

However, this study also has some limitations: ① Sampling from a single city is insufficient to represent the diversity of medical regions in China; ② Even with the Harman test to control for common method bias, the self-assessment data may still be biased; ③ The cross-sectional design cannot infer causal relationships. In future research, further exploration can be conducted on the management strategies that need to balance ideals and reality: ① In the case of manpower shortage, improving the career experience can be achieved through the mentorship system rather than simply reducing the frequency of night shifts; ② When enhancing organizational fit, attention should be paid to the limited autonomy of nurses within the hierarchical system, and low-cost interventions such as departmental culture building should be prioritized. ③ Important covariates, including emotional exhaustion, work environment, complexity of work roles, and leadership style, are not accounted for in the analysis, which may result in confounding effects. In subsequent stages, research accuracy could be enhanced by incorporating these covariates into the analytical framework.

## Data Availability

The original contributions presented in the study are included in the article/supplementary material, further inquiries can be directed to the corresponding author.
